# Design and Characterization of a Novel Blood Collection and Transportation Device for Proteomic Applications

**DOI:** 10.3390/diagnostics10121032

**Published:** 2020-12-02

**Authors:** Nathan K. Kaiser, Maximillian Steers, Charles M. Nichols, Hestia Mellert, Gary A. Pestano

**Affiliations:** Biodesix Inc., 2970 Wilderness Place Suite 100, Boulder, CO 80301, USA; Maximillian.Steers@biodesix.com (M.S.); Charles.Nichols@biodesix.com (C.M.N.); hestia.mellert@biodesix.com (H.M.); gary.pestano@biodesix.com (G.A.P.)

**Keywords:** blood collection device, diagnostics, clinical proteomics, MALDI-ToF, Multiple Reaction Monitoring (MRM)

## Abstract

A major hurdle for blood-based proteomic diagnostics is efficient transport of specimens from the collection site to the testing laboratory. Dried blood spots have shown utility for diagnostic applications, specifically those where red blood cell hemolysis and contamination of specimens with hemoglobin is not confounding. Conversely, applications that are sensitive to the presence of the hemoglobin subunits require blood separation, which relies on centrifugation to collect plasma/serum, and then cold-chain custody during shipping. All these factors introduce complexities and potentially increased costs. Here we report on a novel whole blood-collection device (BCD) that efficiently separates the liquid from cellular components, minimizes hemolysis in the plasma fraction, and maintains protein integrity during ambient transport. The simplicity of the design makes the device ideal for field use. Whole blood is acquired through venipuncture and applied to the device with an exact volume pipette. The BCD design was based on lateral-flow principles in which whole blood was applied to a defined area, allowing two minutes for blood absorption into the separation membrane, then closed for shipment. The diagnostic utility of the device was further demonstrated with shipments from multiple sites (n = 33) across the U.S. sent to two different centralized laboratories for analyses using liquid chromatography/mass spectrometry (LC/MS/MS) and matrix assisted laser desorption/ionization-time of flight (MALDI-ToF) commercial assays. Specimens showed high levels of result label concordance for the LC/MS/MS assay (Negative Predictive Value = 98%) and MALDI-ToF assay (100% result concordance). The overall goal of the device is to simplify specimen transport to the laboratory and produce clinical test results equivalent to established collection methods.

## 1. Introduction

Blood is a common biofluid matrix for research use and clinical diagnostic test development as the information available provides valuable insight into the health of the individual. Blood plasma separation is often the first step in blood-based clinical diagnostic procedures and centrifugation is the gold standard method for plasma separation. However, access to the appropriate centrifuge is often limited at draw sites for commercial specimen acquisition. In addition, maintaining sample integrity of the plasma fraction following centrifugation requires cold-chain logistics. Dried blood spots (DBS) are an attractive option as they eliminate the need for centrifugation and cold-chain logistics and have been amenable to numerous analytical techniques and assays [1,2]. DBS are currently used in newborn screening programs worldwide [3]. DBS with mass spectrometric analysis have had significant interest from the bioanalytical community to support toxicological and therapeutic drug analysis [4,5]. There has also been increased interest from the proteomic community in incorporating DBS into the development of diagnostics [6,7,8,9,10]. However, one of the major issues with DBS in the context of proteomics is variability in hematocrit between individuals which can alter the measured analyte concentrations, thus confounding downstream applications [11,12]. To minimize issues with blood hematocrit, volumetric collection of whole blood followed by analysis of the entire sample has been demonstrated to be effective [13,14]. In addition, devices have been developed that separate the “plasma-like” fraction from whole blood without the need for centrifugation [15,16,17]. Since these devices separate the Red Blood Cells (RBCs) through filtration, the analyte concentration could be altered when compared to plasma generated with centrifugation. Thus, correlation of analyte concentration from dried matrix specimen collection techniques should be compared with traditional sample matrices (blood, plasma, or serum) [18,19]. Numerous publications highlight additional challenges and propose analytical guidelines to ensure accurate and reliable data is produced [20,21,22]. In this study we evaluated the hemoglobin content in the dried plasma region after separation in a Blood Collection Device (BCD) as a confounding residue.

Proteomics has traditionally been dominated by bottom-up workflows which consist of proteolytic digestion of the sample followed by liquid chromatography-mass spectrometry (LCMS) analysis of peptides [23]. The LCMS platform has proven effective for protein identification and quantification. The use of LCMS has been valuable in global proteomics for biomarker discovery and subsequent targeted quantitative proteomics for candidate biomarker verification [24]. Extension of the LCMS platform into the clinical setting has significant advantages over more traditional immunochemistry clinical assays in that the molecule of interest is directly measured. The immunochemical clinical assays measure signals generated secondary to the analyte, thus there is no differentiation in the measurement between the analyte and non-specific binding leading to reduced specificity [25]. However, transfer of the mass spectrometry bottom-up workflow into routine clinical diagnostics has been slow due to non-robust method design, clinical validation, and regulatory requirements [26]. With continually improved instrumentation and more robust workflows, LCMS-based proteomics has the potential to make continued advancement into clinal diagnostics [27]. In addition to LCMS-based platforms, the matrix assisted laser desorption/ionization-time of flight (MALDI-ToF) mass spectrometry platform is a very attractive option for clinical applications due to ease of use, low operating costs, and high throughput which leads to low cost per sample [28,29]. Though the platform does have its own limitations, MALDI-ToF has been successfully implemented for routine microbial species identification in clinical microbiology [30,31]. The use of MALDI-ToF has also shown utility in personalized medicine to stratify patients into responders and non-responders for personalized drug treatment options [32].

Presented here is a novel BCD designed with the intended use for downstream proteomic applications. The device accepts whole blood and separates it into cellular and liquid fractions; the separation of RBCs minimizes hemoglobin abundance in the liquid fraction which can cause confounding results for some applications [13]. The design criteria for the BCD is listed as follows: (1) accepts whole blood as the specimen type, (2) separates the cellular fraction from the liquid fraction, (3) minimizes hemoglobin abundance in liquid fraction, (4) minimizes protein degradation at ambient temperature for at minimum seven days, and (5) provides diagnostic result concordance with currently validated blood-collection methods for commercially available clinical proteomic-based tests [33,34,35]. The ability to provide a low cost, simple to use blood-collection device eliminates the need for field sample centrifugation and frozen plasma transport, which increases availability of clinical proteomic tests to a wider patient population.

## 2. Materials and Methods

### 2.1. Design of Blood-Collection Device

The design of the BCD is based on the principles of lateral flow where whole blood is separated over a membrane into cellular and liquid fractions. The blood separation membrane (LF1, GE Healthcare Bio-Sciences Corp, Piscataway, NJ, USA) in this device is enclosed in a dual hinged cassette. The dual hinge design has one door specific for blood application, and a second door for sample retrieval. To promote even flow of blood along the separation membrane, it lies flat against the bottom of the cassette. A desiccant pack is positioned in the hinged door directly over blood application area. The desiccant promotes quick drying of the whole blood specimen when the cassette is closed, as it was observed that hemoglobin levels increased in the separated liquid fraction the longer the specimen remained wet. The desiccant pack is held in place mechanically to avoid outgassing of adhesive into the separation membrane. The mesh in the blood application area provides a defined region for blood application, stabilizes blood movement while it is absorbed into the separation membrane and distributes blood flow evenly across the width of the device. The length of the separation membrane (85 mm × 17 mm) was optimized such that the fluid portion of the blood will migrate to the end of the device when ~250 μL of whole blood is deposited at one end. A single use disposable exact volume transfer pipet (P/N 139118, Global Scientific, Mahwah, NJ, USA) was used to deposit the blood on the device.

### 2.2. Collection of Whole Blood

Whole human blood was obtained by venipuncture by a licensed medical specialist from three separate cohorts: (1) previously diagnosed with non-small cell lung cancer (NSCLC), (2) patients with existing lung nodules and (3) normal healthy volunteers. Studies were Institutional Review Board (IRB) approved through Advarra IRB (31515, approved on 15 November 2018 and 33493, approved on 22 February 2018) and all patients were consented before any study activities were performed. Normal healthy donors were consented under Biodesix protocol for normal human donors. For blood collection with the BCD for all cohorts, blood was drawn into a 3 mL K2EDTA tube (Greiner Bio-One, Kremsmünster, Austria) and a 250 μL exact volume transfer pipet (Globe Scientific, Mahwah, NJ, USA) provided with the device was used to transfer blood to the BCD. The device remained open for approximately 2 min to allow the blood to absorb into the separation membrane before the cassette was closed and packaged in a moisture tight envelope for overnight delivery. Additionally, for the NSCLC cohort, blood was taken from the same K2EDTA tube with a second transfer pipet, and 3–4 drops of whole blood were placed in the defined application area of a commercially available blood-collection device, defined as “reference” in this study (HemaSpot-SE, Spot on Sciences, San Francisco, CA, USA). Blood taken from the same tube should minimize potential differences with the associated phlebotomy. The device remained open for approximately 2 min to allow the blood to absorb into the separation membrane before the cassette was closed and packaged in a moisture tight envelope for overnight delivery. For the cohort with existing lung nodules, in addition to collection of the BCD, matched plasma samples were obtained and processed through a clinically validated Multiple Reaction Monitoring (MRM) assay [35].

### 2.3. MALDI-ToF Profiling

Specimens were extracted from the BCD separation membrane with a 3 mm punch (Whatman, UK). Unless otherwise noted, three evenly spaced punches were obtained laterally along the center of the separated liquid portion of the device. The punches were typically taken one-two days after whole blood was applied, to allow for shipment of specimen from sites across the country. All thee punched disks were placed in a single 1.5 mL Eppendorf tube and eluted with 50 µL of high performance liquid chromatography (HPLC) grade water (VWR, Radnor, PA, USA) and processed as previously described [34]. Specimens were extracted from the reference device with a single punch and processed in accordance with the clinical assay protocol [34]. In brief, equal volumes (20 μL) of extracted specimen and MALDI sinapinic acid matrix were mixed for 1 min, then 2 μL was manually spotted in triplicate on the sample target and allowed to dry on the bench. MALDI-ToF mass spectra were obtained in linear mode on an AutoFlex Speed (Bruker Daltonics, Billerica, MA, USA) from *m/z* 3000–30,000. A total of 2000 laser shots were collected per spectra (100 shots per laser spot) with the ion intensity threshold feature enabled. Each spectrum was evaluated with built-in quality control (QC) metrics for minimum number of peaks, noise level, and peak alignment, and the spectra were then visually inspected. Spectra were processed through an algorithm that produced a binary classification [33]. Classification labels were only obtained if all three technical replicates agreed.

### 2.4. Multiple Reaction Monitoring

Plasma specimens from the cohort with existing lung nodules were processed through a MRM clinical assay previously described [36,37], and the matched specimen from the BCD was processed through an analogous MRM workflow [38]. Results from both test processes were evaluated for concordance. Briefly, for specimens that were procured with the BCD, an area 1.7 cm wide and 1.5 cm in length was excised from the plasma region of the membrane of the BCD starting approximately 0.5 cm from the end of the RBCs. The decision to remove a section established a repeatable area for protein extraction and minimized the potential for heterogeneity across the membrane. The section was quartered and placed into a 0.45 μm centrifugal spin filter (VWR, Radnor, PA, USA), 80 µL of PBS was added and vortexed for 5 min. The sample was centrifuged for 2 min at 12,000× *g*. High abundance proteins were removed with depletion HPLC utilizing an Agilent 1260 HPLC with a Multiple Affinity Removal System (MARS) Column Human 14 (Agilent, Santa Clara, CA, USA). Depleted samples were enzymatically digested with 33 µL of 0.5 mg/mL trypsin (Worthington Biochemical Corp., Lakewood, NJ, USA) for 4 h at 37 °C on an orbital shaker (250 RPMs). Following digestion, Stable Isotope-labeled Standards (SIS) peptides (New England Peptide, Gardner, MA, USA) were added, and the specimen was purified with Solid Phase Extraction (SPE) using a C18 96-well solid phase extraction plate (Tecan, Männedorf, Switzerland). Samples were eluted by adding 200 µL 48/52 (acetonitrile/0.1% TFA in water). The samples were split evenly into two separate 96-well plates and evaporated to dryness overnight. The dried samples were then reconstituted with 12 µL 10/90/0.1% acetonitrile/water/formic acid. A HPLC (1290) coupled to a triple quadrupole mass spectrometer (6490) was used for sample analysis (Agilent, Santa Clara, CA, USA). Chromatographic peak areas of the quantitative transitions were determined using MassHunter Quantitative Analysis software (version B.05.00, Agilent, Santa Clara, CA, USA).

## 3. Results and Discussion

### 3.1. Whole Blood Separation

**Figure 1:**
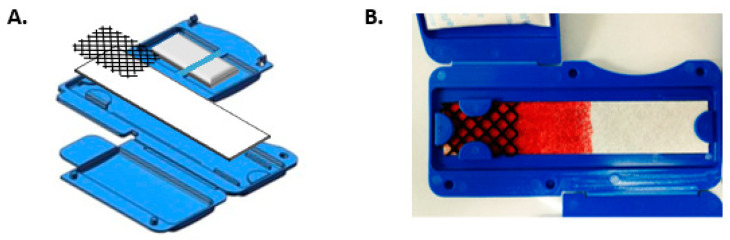
**A.** Schematic illustration of the blood-collection device. **B.** Representative separation membrane after application of whole blood showing separation of the red blood cells from the liquid portion of whole blood.

The device was designed to accept whole blood and to separate the cellular and fluid fractions. A conceptual design of the BCD is shown in Figure 1.

The separation membrane impedes the flow of larger particles while the soluble components flow freely throughout the device. Figure 1 shows the separation of whole blood that occurs in the device. There is clear separation between the red blood cells and the liquid portion of the blood. It is typical for the red blood cells to travel approximately halfway across the device while the fluid fraction migrates to the end of the strip. However, the exact travelled distance will depend on the hematocrit level and blood viscosity of the specimen. Though the device does not require addition of accurate blood volume for the presented applications, use of the exact volume transfer pipet removes the variability associated with the application of drops of blood. Based on total protein concentration, it was determined that a 1 cm^2^ section from the fluid fraction of the device is equivalent to 7.8 μL of plasma with a coefficient of variation of 21% (Supplementary Figures S1 and S2). The experimental design of the assays investigated account for the variability in eluted protein content by measuring relative protein abundance with MALDI-ToF assay or peptide ratios with the MRM assay. The ability of the BCD to separate the blood into its components reduces sample preparation time and complexity for the health care professional performing specimen collection.

### 3.2. Spectral Hemoglobin

**Figure 2:**
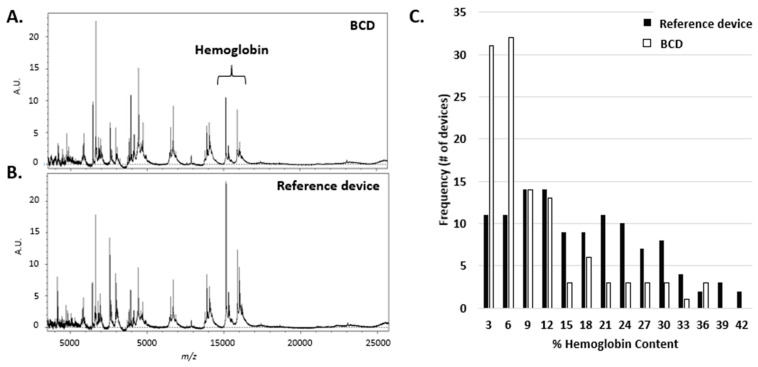
Typical matrix assisted laser desorption/ionization-time of flight (MALDI-ToF) mass spectra of the separated liquid fraction obtained from **A.** the blood-collection device (BCD) and **B.** Reference device (HemaSpot-SE). **C.** Histogram of the percentage spectral hemoglobin content for each of the devices (n = 112, bin size is 3%) obtained in the concordance study. The histogram counts the number of devices that were received that fall within a defined range of spectral hemoglobin content.

Significant amounts of free hemoglobin in plasma may occur following disruption of red blood cells (RBCs). Normal blood draw procedures cause a limited degree of unavoidable disruption and may result in small amounts of free hemoglobin. The free hemoglobin is likely to increase with a traumatic blood draw or prolonged exposure of the non-cellular milieu to red blood cells post-draw as they lyse and release hemoglobin. For MALDI-ToF profiling experiments, which rely on the qualitative peak heights in the spectrum, if the amount of free hemoglobin is excessive it will dominate the spectrum, suppressing less abundant species and limiting the dynamic range of the assay. Thus, if the RBCs are disrupted before plasma separation, the hemoglobin peaks become significant and can result in quality control failures and uninterpretable test results. Figure 2 displays the average spectrum obtained with the BCD compared to the reference device. Both the *α* and *β* subunits of hemoglobin are observed at *m/z* 15,127 and *m/z* 15,868, respectively. It is not uncommon for the hemoglobin peaks to be among the most abundant peaks observed in the spectrum when the reference device is used for specimen collection as shown in Figure 2B.

A prospective field concordance study of NSCLC patients was performed with 112 matched specimens collected with both the BCD and a reference device. The reference device has been previously validated as an acceptable blood-collection device for the MALDI-ToF clinical assay and is in current commercial use. A histogram for both devices that shows the distribution of hemoglobin content is shown in Figure 2C.

The spectral hemoglobin content is calculated as the percentage of the integrated area of the hemoglobin peaks relative to the total integrated intensity of all peaks in the spectrum. Each device in the study was binned based on the total spectral hemoglobin content. With the BCD, many devices exhibited low spectral hemoglobin content with the majority of the devices falling within the first two bins (0%–3% and 3%–6%). The reference device had a broader distribution of hemoglobin content when compared to the BCD. Overall, there was an approximate 40% reduction in the average spectral hemoglobin content with the BCD, with a median reduction of 55%. In the study, where devices are being spotted in the field by phlebotomists and not in the laboratory, one reference device was rejected during initial receipt of the device due to hemolysis and an additional three samples processed from the reference devices were rejected upon visual inspection of the spectra due to excessive hemoglobin content. In contrast, none of the samples processed from the BCD failed due to excessive spectral hemoglobin content.

### 3.3. Protein Concentration Gradient

**Figure 3:**
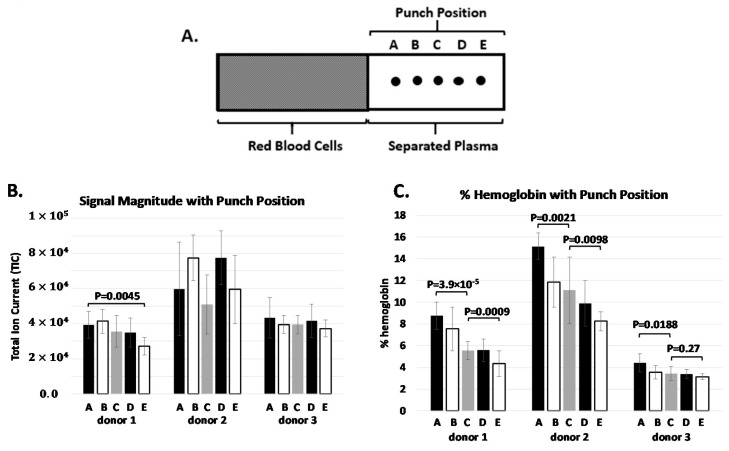
**A.** graphical representation of the whole blood separation strip and location of the punch position in the liquid portion. **B.** Total ion current at each of the punch positions for three different donors. Each bar is the average of nine spectra. **C.** The hemoglobin content in the spectra at each of the punch positions. P-values were calculated between positions (A and C) and (C and E). Results show a clear decrease in the hemoglobin content for each of the donors the further the punch is taken from the red blood cells (RBCs).

Following plasma separation from whole blood on the BCD, approximately half the separation membrane is available for specimen extraction. To characterize the uniformity of the protein content across the separation membrane, multiple punches (5) were taken laterally across the device as shown in Figure 3. Three normal healthy donors, three devices spotted per donor, five punches per device were analyzed individually and ran in triplicate for a total of 135 measurements. The total spectral intensity and spectral hemoglobin content was recorded at each punch position and results are shown in Figure 3.

There is a slight observable trend in total spectral intensity content across the separation membrane for donor 1 and none for donors 2 and 3. Overall, the difference in total protein laterally across the separation membrane is likely lower than the variability associated with MALDI signal generation. Perhaps more importantly, each sampling location across the membrane (A though E) produced a valid diagnostic label when processed using the clinical MALDI-ToF assay, indicating that the entire separated liquid fraction has sufficient protein content available for analysis. In contrast to total protein content, the spectral hemoglobin content showed a consistent decreasing trend the further from the RBCs the sample was procured, and this was true across all three donors. The *p* values between select sample locations are shown in Figure 3.There is a significant difference in spectral hemoglobin content across the separation membrane for both donors 1 and 2. For donor 3 there was a significant difference between the first and middle locations, but no difference between the middle and end locations, likely to be due to the low initial spectral hemoglobin content (~2–3%). These concentrations of hemoglobin observed for donor 3 are representative of samples generated through centrifugation-based methods with minimal RBC disruption. Thus, we would not expect to see values much lower. There was no observed correlation between the total ion current and the hemoglobin content. One likely explanation for the observed hemoglobin gradient is that a few of the RBCs continue to rupture during the lateral separation of phases - when rupture happens early in the process, the hemoglobin will migrate the full length of the strip, while those RBCs that rupture after the liquid has migrated to the end of the strip will result in minimal hemoglobin migration. Once the strip is dried the hemoglobin will be fixed, hence it becomes important to dry the sample quickly after sample separation to minimize hemoglobin movement along the strip. To improve specimen drying time and capacity an additional desiccant pack is placed within the shipment pouch. For the clinical assay, punches are typically procured around locations B-D in Figure 3A to capture potential variation in protein concentration while minimizing spectral hemoglobin content.

### 3.4. Specimen Stability

One design criterion for the device is the facilitation of field collection of patient specimens and shipment to a centralized testing facility for analysis. Therefore, the specimens need to remain stable within an appropriate temperature range for transport. To test specimen stability for the MALDI-ToF clinical assay, donor whole blood specimens were collected from two healthy individuals by a licensed phlebotomist; each donor’s blood was spotted onto 20 BCDs, sealed individually in a desiccant pouch, and allowed to dry for 2 h. Four BCDs from each donor were then incubated under each of the following conditions before analysis: −20 °C (18 h), ambient temperature (18 h), and 40 °C (2, 6 and 18 h). For the MALDI-ToF assay, which generates a binary classification result [33], all specimens at all time points and temperatures generated a clinically valid classification test result, (see Table 1). The results of the analysis ‘pass’ when all four BCDs from each temperature/time point produce the same clinical classification.

**Figure 4:**
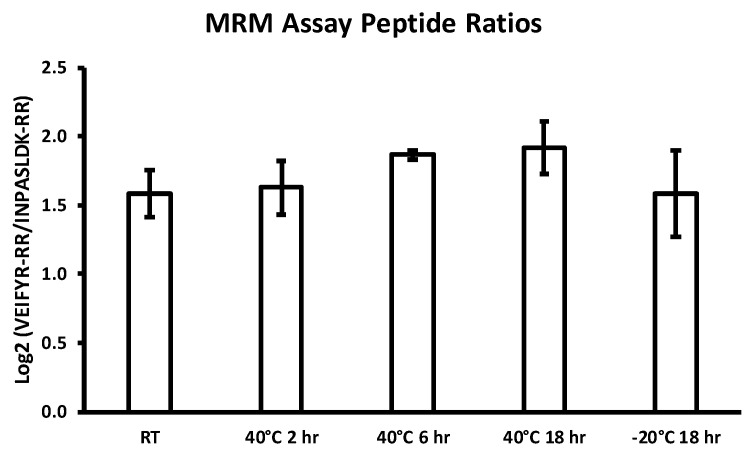
Peptide ratios measured by the Multiple Reaction Monitoring (MRM) assay for specimens applied to the BCD, dried and subjected to incubation at extreme environmental temperatures that may be encountered during transport. The error bars represent one standard deviation from the mean.

For the MRM assay, the same temperatures and time points were investigated (four BCDs each) for a single donor. The results are shown in Figure 4. The peptide ratios for all conditions appeared to be similar, and there was no significant difference observed. For both proteomic assays investigated, the specimens of interest were determined to be stable on the BCD over the temperature range and duration evaluated. Thus, the stability results indicate that the BCD is well suited for in-field collection and transport of blood specimens to testing facility.

To test specimen stability over an extended period, whole blood samples were procured from three consented normal heathy donors by a licensed phlebotomist. Ten BCDs per donor were spotted and sealed individually in desiccant pouches and stored on the bench at ambient temperature. One BCD from each individual was analyzed by MALDI-ToF each day and processed through the clinical test workflow, starting the day after the device was spotted. The BCD was evaluated on the ability to produce a clinically valid diagnostic test result. Spectral intensity was also monitored for specimen degradation. It was expected that as the specimen degrades the total spectral intensity would decrease. Thus, if degradation had occurred the spectra would fail to be collected (below signal intensity threshold) and fail the QC criteria or visual inspection. In this study, all specimens across all analysis time points produced a clinically valid test result. Therefore, the specimen remains stable on the BCD for extended time periods at ambient temperature, which is important if the initial analysis of a clinical specimen produces an invalid result and needs to be reanalyzed. The total spectral intensity for each specimen in the 10-day stability study did not show a significant trend in reduction of ionizable protein over the course of a week (Supplementary Figure S3). The MALDI-ToF assay is a qualitative test which relies on the relative ratio of spectral abundance of proteins. Therefore, even if the total spectral intensity changes, the protein ratios should remain consistent (Supplementary Figure S3). The whole protein specimen on the BCD remained stable, in that clinically valid test results were generated for the duration of the 10-day study.

### 3.5. MALDI-ToF Assay Label Concordance

The reference-collection device was previously validated for whole blood specimen collection and transport for the multi-variate proteomic MALDI-ToF mass spectrometry-based assay in our Clinical Laboratory Improvement Amendments (CLIA) testing laboratory. The qualitative assay assigns a binary classification of ‘Good’ (i.e., identifies patients more likely to respond to standard treatments) or ‘Poor’ (i.e., indicates chronic inflammation and an aggressive disease state) to serum or plasma samples. The Poor classification has been shown to be associated with increased levels of acute phase reactants and inflammation [39,40]. These classifications can be used to predict which patients will experience longer survival, independent of patient performance status, other biomarker status, treatment of choice or line of therapy [34,41,42]. The commercial assay has been interrogated retrospectively and prospectively in samples for randomized trials that confirm the prognostic information associated with the protein profile [43]. Here we demonstrate equivalent clinical results between the BCD and the defined validated reference-collection device. Matched specimen samples were collected from various sites (n = 24) across the United States and shipped to the centralized laboratory for testing.

This study evaluated 112 NSCLC donors with matching specimens. 100 of these specimens were used to assess concordance of the resultant clinical classification between the BCD and reference device. Twelve specimens were excluded from the study because either one or both devices failed to produce agreement within triplicate runs and were deemed “indeterminate” (5/112), or spectra failed to acquire or pass QC parameters (7/112). The failure rate was 3.6% (4/112) and 4.5% (5/112) respectively for the BCD and reference device, 2 specimens failed for both devices. Results for the concordance study are shown in Table 2**.** In summary, we observed 100% result concordance between the two specimen collection devices. The equivalent results generated for specimens collected on the novel BCD and the reference device signify that the device is acceptable for use in clinical proteomic tests. In conclusion, the reduced hemoglobin levels presented with the BCD minimizes the probability of sample rejection while still maintaining accurate results with the predicate device. Because sample rejection leads to the need for additional blood draws, it necessarily increases time to actionable results. Fewer QC failures means faster turnaround of results and better overall support for patients and physicians as they make critical treatment decisions.

### 3.6. Multiple Reaction Monitoring Concordance

**Figure 5:**
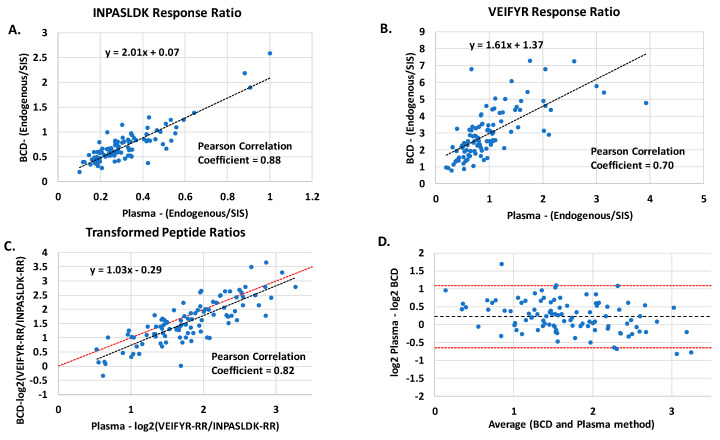
Peptide comparison between the BCD-collection method and plasma-collection method **A**. RR of peptide INPASLDK **B**. RR of peptide VEIFYR. **C**. Comparison of the peptide ratios RR for the two methods after the applied correction factor. The dotted red line represents the identity line. **D**. The corresponding Bland-Altman Plot for the method comparison, the red dotted line represents the 95% confidence internal.

The BCD was also incorporated into a bottom-up proteomics MRM assay for peptide quantification. The BCD was included in a clinical assay that incorporates the ratio of relative abundance of plasma proteins in circulation along with clinical factors to evaluate the probability of lung nodules being benign [35,37,44]. The advantage of incorporation of peptide ratios as opposed to absolute peptide quantitation is that pre-analytic variables, as well as variation in the depletion and digestion steps, are accounted for [44]. The two plasma proteins that were measured are Galectin-3-binding protein (LG3BP) using target peptide VEIFYR and Scavenger receptor cysteine-rich protein (C163A) using target peptide INPASLDK. With our focus on pre-analytics, the objective was to determine whether equivalent diagnostic results could be obtained from the frozen-shipped plasma and the ambient-shipped BCD. Since the clinical results of the assay may rule out the likelihood that a nodule is malignant, the acceptance criteria for comparison of the two assays was the Negative Predictive Value (NPV) (i.e., a false negative result would classify the nodule as a high probability of being benign when actually it was malignant). The comparison of the two workflows with matched specimens that were collected as either plasma or BCD is shown in Figure 5. In Figure 5, the Response Ratio (RR) is measured by taking the ratio of the chromatographic peak area of the endogenous by the chromatographic peak area of the SIS peptide.

The Pearson correlation coefficients for the two measured peptides were 0.88 for INPASLDK and 0.70 for VEIFYR. The MRM assay was originally developed and validated with the plasma collection method. To normalize peptide ratios from the BCD method for use in the test algorithm, a scaling factor is utilized. Comparison of the peptide ratios before the scaling factor is applied is shown Supplemental Figure S4. Using a scaling factor to adjust for differences in peptide abundances between the BCD-collection and plasma collection methods revealed good correlation of clinical test results, which also incorporate a number of clinical factors, between the two workflows (NPV agreement = 98%). Comparison of the ratio of the two peptides (VEIFYR-RR/INPASLDK-RR) between the two collection methods gave a Pearson correlation coefficient of 0.82, as shown in Figure 5C. The identity line shown in the figure represents perfect agreement between methods, with a slope of 1 and an intercept of 0. The Bland-Altman Plot in Figure 5D is displayed to show potential bias between the methods; ideally the average difference should approach 0. The average difference between methods was 0.22, and the value has a magnitude of approximately 0.5 standard deviations. With the NPV in high agreement between the plasma and BCD measurements, the slight bias has no significant impact in context of the assay. With the slope of the corrected peptide ratios approaching ideal, a slight modification to the correction factor would eliminate the bias. This study established that the BCD may be successfully incorporated into this second type of mass spectrometric clinical assay that utilizes a quantitative MRM workflow. Direct comparison of protein abundance from the BCD or other type of card-based collection method with frozen plasma specimens could result in altered measured abundances and may require correction factors.

## 4. Conclusions

A novel whole blood-collection device that separates plasma from cellular components designed for downstream mass spectrometry applications is presented. The BCD utilizes the principles of lateral flow where the separation membrane impedes larger cellular components while allowing the liquid fraction to flow freely through the device. The specimen is dried on the separation membrane and may be transported under ambient conditions to clinical laboratories. The separated plasma can then be resuspended and used directly in the mass spectrometry workflow. The device is designed to minimize the amount of hemoglobin in the plasma fraction resultant from rupture of red blood cells, which can cause inaccurate results or prevent valid test results altogether. As a proof of principle, plasma proteins were eluted from the separation membrane with water and directly used in a clinically validated MALDI-ToF test without any additional sample clean up. There was 100% concordance in clinical diagnostic labels between patient samples collected with the BCD and a current approved collection device for the test. Additionally, the collected proteins were stable over the course of 10 days while being stored at ambient temperature, and between the temperatures −20–40 °C for 18 h. The second clinical test example utilized an MRM-based assay. Specimens were eluted from the separation medium and subjected to a bottom-up proteomic workflow. Results from 101 matched plasma and BCD specimens revealed good correlation between the two sample collection methods (NPV agreement = 98%) when a scaling correction factor for peptide abundances was applied. For both assays, the good correlation of clinical test results between the BCD and previously validated collection methods indicate that the BCD specimen collection method is suitable for clinical specimen collection.

The ease of use of the BCD for separation and transport of biological whole blood specimens makes it ideal for clinical diagnostics. Here we demonstrate that the device is compatible with both MALDI-ToF and MRM blood-based proteomic assays that were investigated here. However, the BCD could also be applicable as a collection and transport device for other diagnostic tests that utilize blood-based specimens (e.g., ELISA or nucleic acid testing). In this study, blood was collected through venipuncture, but slight modifications such as reduction in device size could make it a compelling option for collection of capillary blood utilizing a figure prick for blood extraction. Modification of the device could result also in its use for lateral flow in rapid point of care testing.

In conclusion, the BCD enables specimen collection for prescribed clinical tests to be collected in the field, including at the patient’s home, by a trained phlebotomist, eliminating the need for the patient to go to a clinic. The collected specimen is shipped at ambient temperature in a barrier pouch, alleviating the need to arrange for dry ice or other cold chain transport, and thus reducing transport logistics and shipment costs. The simplicity of the device has the potential to expand the patient testing options for blood-based proteomic tests.

## Figures and Tables

**Table 1:** Results from the temperature stability study for the MALDI-ToF assay.

Temperature	Duration	Donor 1	Donor 2
Ambient	18 h	Pass	Pass
−20 °C	18 h	Pass	Pass
40 °C	2 h	Pass	Pass
40 °C	6 h	Pass	Pass
40 °C	18 h	Pass	Pass

**Table 2:** Concordance in classification of clinical results for the BCD and the reference device.

	Reference Good	Reference Poor
**Test (BCD)** **Good**	85	0
**Test (BCD)** **Poor**	0	15

## Supplementary Materials

The following are available online at https://www.mdpi.com/2075-4418/10/12/1032/s1, Figure S1: BCD sectioning for elution studies, Figure S2: Total protein elution, Figure S3: protein ratio from 10-day stability study, Figure S4: Peptide ratios before applied correction factor.
