# Supplementary material for: Design and Characterization of a Novel Blood Collection and Transportation Device for Proteomic Applications
Source: Diagnostics (Basel). 2020 Dec 2;10(12):1032. doi: 10.3390/diagnostics10121032 (PMC7761483; doi:10.3390/diagnostics10121032)
Supplement: Supplementary file 1 [file diagnostics-10-01032-s001.pdf]

Supplementary Data:

Protein Elution Studies:

With the BCD the amount of protein measured will be a function of area as compared to typical volume with plasma. Therefore, the area of the device that would be needed for elution to get an equivalent amount of protein per volume of plasma needs to be known.

BCDs spotted with whole blood and matched plasma were compared.

The 3 cm of the separated plasma portion of the BCD devices (26 devices per day) were sectioned into 8ths, see **Supplementary figure 1**. Then all 8 sections were placed in a single 0.45 µm centrifugal spin filter (VWR, PA) and 160 µL of water added, then vortexed for 10 minutes at 1,000 RPMs, and centrifuged (12,000 g) for 2 minutes. Then a Qubit fluorometer (Qubit 3.0, Thermo Fisher Scientific) was used, per protocol recommended by the manufacturer, to determine the concentration of the eluate and compared to the concentration of the matched plasma also determined by Qbit.

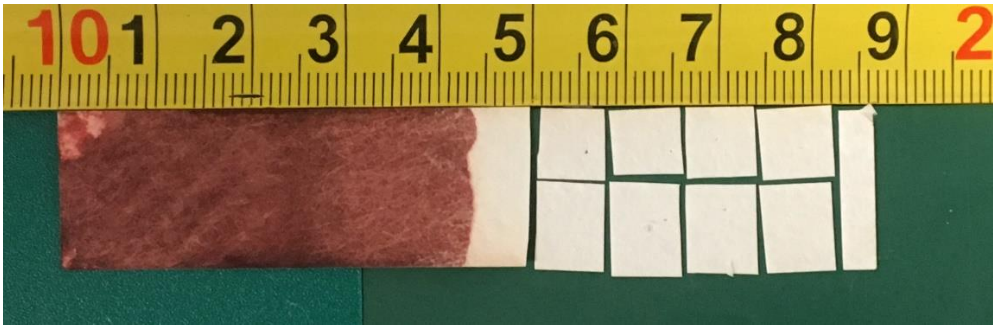

Figure S1: Picture of the separated plasma and the sectioned paper used for the protein elution studies.

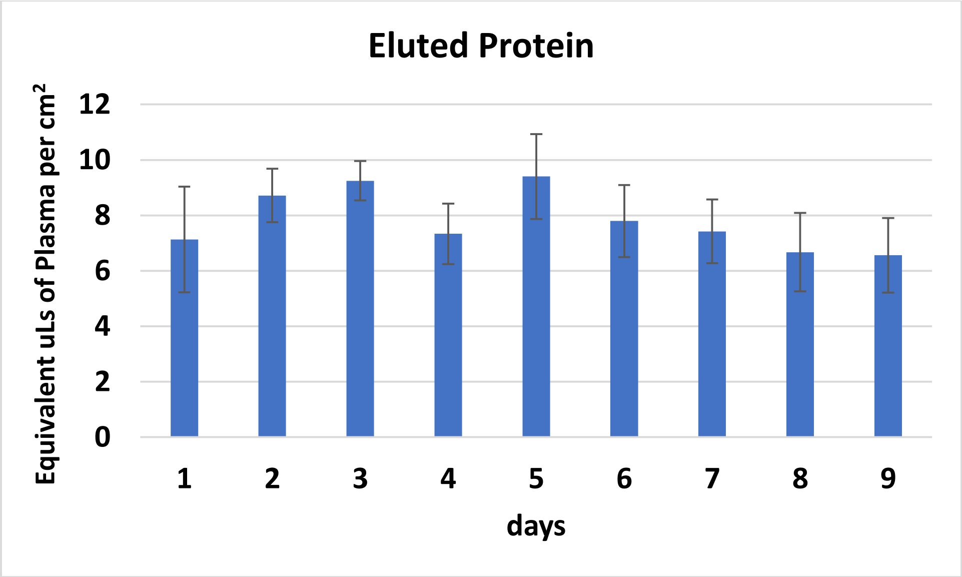

Figure S2. Plasma volume equivalent / cm^2 based on Qbit measurements. The error bars represent 1 standard deviation. 26 BCDs are measured on each of the days.

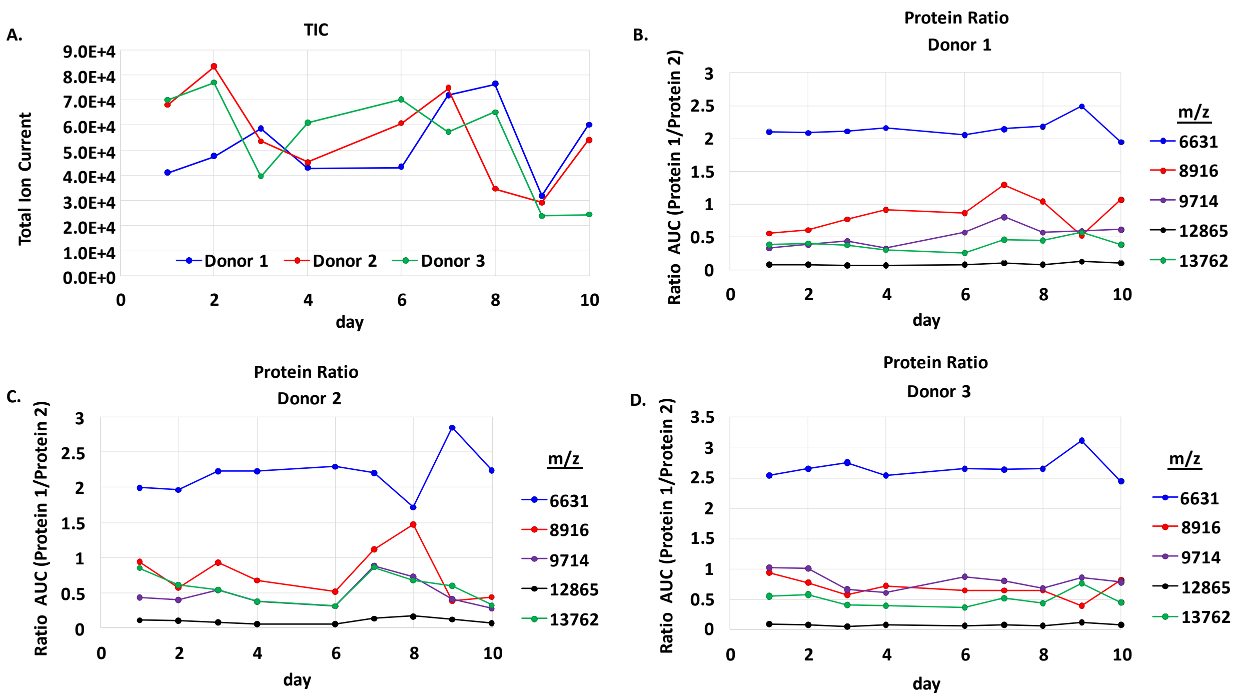

Figure S3. Results from the 10-day stability study with the MALDI-ToF assay. A. The Total Ion Current for each of the donors. Each point is the average of 3 analytical replicates of a single BCD. B-D. Protein ratio for different proteins present in the spectra. Each protein is normalized to the Area Under Curve (AUC) of Apolipoprotein C-I (29-83), the AUC for each of the m/z is predominantly the following species: m/z 6631 –Apolipoprotein C-I (27-93) m/z 8916 – Apolipoprotein C-II, m/z 9714 – Apolipoprotein C-III, m/z 12865 -Serum Amyloid A4, m/z 13762 -Transthyretin . The protein ratios remain consistent over the course of the 10-day stability investigation.

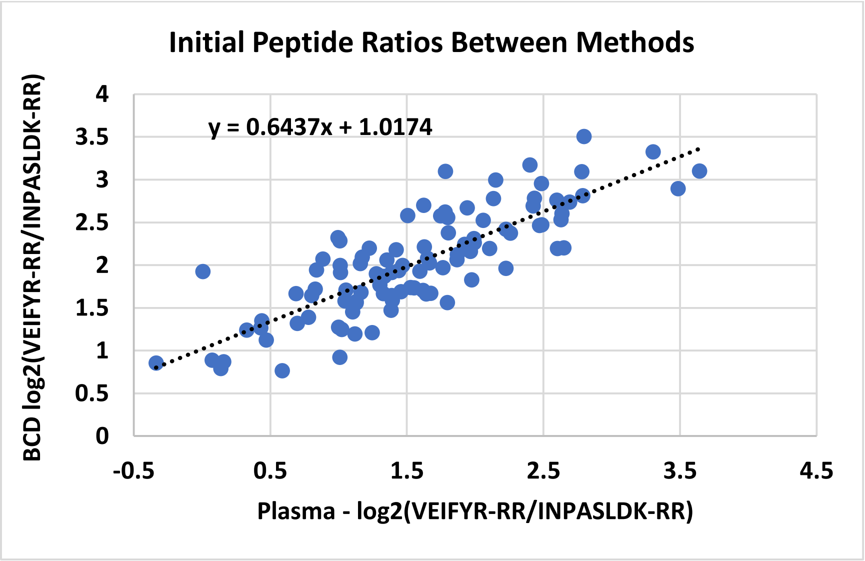

Figure S4. Peptide ratio comparison between methods before the correction factor is applied.
